# Supplementary material for: Reconciling Mining with the Conservation of Cave Biodiversity: A Quantitative Baseline to Help Establish Conservation Priorities
Source: PLoS One. 2016 Dec 20;11(12):e0168348. doi: 10.1371/journal.pone.0168348 (PMC5173368; doi:10.1371/journal.pone.0168348)
Supplement: S1 Dataset — (ZIP) [file pone.0168348.s002.zip › Taxa/Serra Norte/SN_2013/N5S/Lista N5S-05.pdf]

CAVIDADE N5S-0005

Classe	Ordem	Fam/Outros	Gên/Outros	Espécie	S-U	U-S	Zon.
Arachnida	Acari	Bdellidae		sp.1	X		E
Arachnida	Acari	Trombidiidae		sp.67	X		E
Arachnida	Acari			jovem	X	X	
Arachnida	Amblypygi	Phrynidae	*Heterophrynus*	*longicornis*	1	X	
Arachnida	Araneae	Ctenidae		jovem	3		E/P
Arachnida	Araneae	Drymusidae	*Drymusa*	jovem		X	
Arachnida	Araneae	Filistatidae		sp.1	X		E/P
Arachnida	Araneae	Mysmenidae	*Microdipoena*	sp.1	X		E
Arachnida	Araneae	Ochyroceratidae	*Ochyrocera*	sp.2	X		E
Arachnida	Araneae	Pholcidae	*Leptopholcus*	sp.1	X		E
Arachnida	Araneae	Pholcidae	*Mesabolivar*	aff. *cambridgei*	X		P
Arachnida	Araneae	Pholcidae	Ninetinae	sp.1	X		E/P
Arachnida	Araneae	Pholcidae		jovem	X	X	E/P
Arachnida	Araneae	Salticidae		sp.7	X	X	E
Arachnida	Araneae	Scytodidae	*Scytodes*	sp.1	14	X	E/P
Arachnida	Araneae	Sparassidae		jovem	X		P
Arachnida	Araneae	Tetragnathidae		jovem		X	
Arachnida	Araneae	Theraphosidae		jovem	3	X	E/P
Arachnida	Araneae	Theridiidae	*Theridion*	sp.1	X	X	E
Arachnida	Araneae	Theridiosomatidae	*Plato*	sp.1	X		P
Arachnida	Araneae	Uloboridae		jovem		X	
Arachnida	Opiliones	Stygnidae	*Protimesius*	*gracilis*	1		P
Arachnida	Pseudoscorpiones	Chernetidae	*Spelaeochernes*	sp.1	X	X	E/P
Arachnida	Pseudoscorpiones	Chthoniidae		jovem	X		E
Arachnida	Pseudoscorpiones	Olpiidae		jovem	X	X	E
Chilopoda	Scutigeromorpha	Pselliodidae	*Sphendononema*	jovem	1		E
Entognatha	Collembola	Isotomidae		sp.1	X		P
Entognatha	Diplura	Campodeidae		sp.1	X	X	E/P
Insecta	Blattodea	Blattellidae		sp.1	4		E
Insecta	Blattodea	Blattidae		sp.5	7		E/P
Insecta	Blattodea	Polyphagidae		sp.1	2		E
Insecta	Blattodea			jovem	11	X	E/P
Insecta	Coleoptera	Nitidulidae		jovem		X	
Insecta	Coleoptera	Scydmaenidae		sp.2	X		E
Insecta	Coleoptera	Staphylinidae	Pselaphinae	sp.18	X		P
Insecta	Diptera	Cecidomyiidae	Cecidomyiinae	sp.	X		E
Insecta	Diptera	Culicidae	*Coquillettidia*	*lynchi*		X	
Insecta	Diptera	Culicidae	*Culex*	sp.	X		E/P
Insecta	Diptera	Drosophilidae		jovem		X	
Insecta	Diptera	Ephydridae		sp.	X		P
Insecta	Diptera	Nematocera		sp.		X	
Insecta	Diptera	Psychodidae	Phlebotominae	sp.	X		E/P
Insecta	Diptera	Sciaridae		sp.	X		E
Insecta	Heteroptera	Dipsocoroidea		sp.	X		E/P
Insecta	Heteroptera	Reduviidae	Emesinae	sp.	1		P
Insecta	Heteroptera	Reduviidae	Reduviinae	sp.	8	X	E/P
Insecta	Homoptera	Cixiidae		jovem	X	X	E/P
Insecta	Hymenoptera	Formicidae	*Acropyga*	sp.1		X	
Insecta	Hymenoptera	Formicidae	*Brachymyrmex*	sp.1	X		E/P

Insecta	Hymenoptera	Formicidae	*Ectatomma*	sp.		X	
Insecta	Hymenoptera	Formicidae	*Hypoponera*	sp.1	X		P
Insecta	Hymenoptera	Formicidae	*Octostruma*	sp.		X	
Insecta	Hymenoptera	Formicidae	*Paratrechina*	sp.1	X		E/P
Insecta	Hymenoptera	Formicidae	*Paratrechina*	sp.2	X		P
Insecta	Hymenoptera	Formicidae	*Pheidole*	sp.1	X	X	E
Insecta	Isoptera	Termitidae	*Nasutitermes*	sp.	X	X	
Insecta	Isoptera	Termitidae	*Subulitermes*	sp.	X		
Insecta	Lepidoptera	Douglasiidae		sp.	X		P
Insecta	Lepidoptera	Noctuoidea		sp.5	2		E
Insecta	Lepidoptera	Tineoidea		sp.1	X	X	P
Insecta	Orthoptera	Acrididae		sp.	1		E
Insecta	Orthoptera	Gryllidae		sp.	6		P
Insecta	Orthoptera	Phalangopsidae	*Paraclodes*	jovem	9		P
Insecta	Orthoptera	Phalangopsidae	*Phalangopsis*	sp.	1	X	
Insecta	Psocoptera			jovem	X	X	E/P
Insecta	Thysanura	Nicoletiidae		sp.1	X		P
Gastropoda	Pulmonata	Systrophiidae	*Happia*	sp.		X	
Malacostraca	Decapoda			sp.		1	
Malacostraca	Isopoda	Philosciidae		sp.		X	
Amphibia	Anura	Strabomantidae	*Pristimantis*	cf. *fenestratus*	2		P
Mammalia	Chiroptera	Emballonuridae	*Peropteryx*	*kappleri*	10	X	E/P
Mammalia	Rodentia	Cricetidae	*Rhipidomys*	sp.	1		E
